# Ultraviolet photoconductive devices with an n-GaN nanorod-graphene hybrid structure synthesized by metal-organic chemical vapor deposition

**DOI:** 10.1038/srep10808

**Published:** 2015-06-01

**Authors:** San Kang, Arjun Mandal, Jae Hwan Chu, Ji-Hyeon Park, Soon-Yong Kwon, Cheul-Ro Lee

**Affiliations:** 1 Semiconductor Materials Process Laboratory, School of Advanced Materials Engineering, Engineering College, Research Center for Advanced Materials Development (RCAMD), Chonbuk National University, Baekje-daero 567, Jeonju 561-756, Republic of Korea; 2 School of Materials Science and Engineering, Low Dimensional Carbon Materials Center, Ulsan National Institute of Science and Technology (UNIST), UNIST-gil 50, Ulsan 689-798, Republic of Korea

## Abstract

The superior photoconductive behavior of a simple, cost-effective n-GaN nanorod (NR)-graphene hybrid device structure is demonstrated for the first time. The proposed hybrid structure was synthesized on a Si (111) substrate using the high-quality graphene transfer method and the relatively low-temperature metal-organic chemical vapor deposition (MOCVD) process with a high V/III ratio to protect the graphene layer from thermal damage during the growth of n-GaN nanorods. Defect-free n-GaN NRs were grown on a highly ordered graphene monolayer on Si without forming any metal-catalyst or droplet seeds. The prominent existence of the undamaged monolayer graphene even after the growth of highly dense n-GaN NRs, as determined using Raman spectroscopy and high-resolution transmission electron microscopy (HR-TEM), facilitated the excellent transport of the generated charge carriers through the photoconductive channel. The highly matched n-GaN NR-graphene hybrid structure exhibited enhancement in the photocurrent along with increased sensitivity and photoresponsivity, which were attributed to the extremely low carrier trap density in the photoconductive channel.

Recently, semiconductor-graphene hybrid-heterostructures^1^,^2^,^3^,^4^ have been actively and extensively studied with the purpose of producing nanoscale semiconductor devices with new forms and functions, enhanced performance, and potential for low-cost mass production. Among the diverse nanostructures being studied, semiconductor nanorods (NRs)^5^,^6^,^7^, and GaN nanorods, in particular, have drawn much attention because of their outstanding features such as their wide direct band gap (3.4 eV), high thermal conductivity (1.3 Wcm^−1^K^−1^), high saturated electron velocity, and good physical and chemical stability; these properties make them ideal for improving the performance of semiconductor devices^8^,^9^,^10^,^11^,^12^.

Graphene^13^,^14^,^15^, known as a novel material by scholars, exhibits superior electrical conductivity^16^ and can serve as an excellent transport medium for charge carriers when integrated with semiconductor heterostructures^1^,^2^,^3^,^4^. Semiconductor NR-graphene hybrid-heterostructures offer large photocurrents and fast recovery^2^. Thus, these structures are ideal for use in high-efficiency optoelectronic devices. Efforts to resolve the thermal issues of light-emitting diodes (LEDs) are ongoing^17^. For LEDs consisting of GaN NR-graphene heterostructures, the semiconductor chip temperature can be reduced by dispersing the heat via the high thermal conductivity of graphene^18^,^19^. Thus, the lifetime, thermal stability and reliability of the LED devices are improved. Low-cost solar cells that contain less semiconductor material can be produced using these GaN NR-graphene heterostructures. Moreover, in the case of solar cells, the high charge carrier mobility of graphene helps collect the photogenerated carriers much more efficiently^4^,^20^,^21^,^22^.

With the expansion of the white LED industry and keeping in mind the importance of GaN NR-graphene hybrid structures, as previously discussed, the most cost-effective technique for producing GaN LEDs without compromising their performance needs to be determined. The vapor-liquid-solid (VLS) technique^23^ using metal-organic chemical vapor deposition (MOCVD)^24^ facilitates nanorod formation with precise growth control and mass production. It is also the most cost-effective technique for growing GaN nanorods. In general, GaN nanorods are grown via the introduction of a metal-catalyst or seed crystal^25^,^26^. Thus, there remains a chance of crystal-contamination in the heterostructures caused by the metal-catalyst^27^. In addition, although graphene exhibits high chemical stability, it is very difficult to form a hybrid-heterostructure by integrating it with a semiconductor material^15^. Even if a hybrid structure is formed, the salient features of the original materials are seriously undermined. Thus, ensuring the prominent presence of the graphene layer in an NR-graphene hybrid structure upon completion of its growth without harming the structural and material properties of the nanorods has remained a challenge for researchers.

In the present study, we sought to form a GaN NR-graphene hybrid structure using the MOCVD technique while maintaining the original properties of the GaN nanorods and graphene. The material and electrical features of this integrated heterostructure were analyzed to assess their potential for developing high-efficiency photoconductive devices. Moreover, the growth of this hybrid structure was achieved on a bare Si substrate without forming silicon dioxide or silicon nitride; furthermore, to avoid contamination, no metal-catalyst was used to grow the GaN NRs. The aim of this study was to demonstrate the highly matched co-existence of well-formed monolayer graphene and GaN NRs in a hybrid structure grown on a Si substrate without using any metal catalyst utilizing the MOCVD process to minimize the production costs. The realization of a high-performance ultraviolet photoconductive device using this hybrid structure was another purpose of this work.

## Results

### Growth and characteristics of monolayer graphene transferred to Si (111)

**Figure 1:**
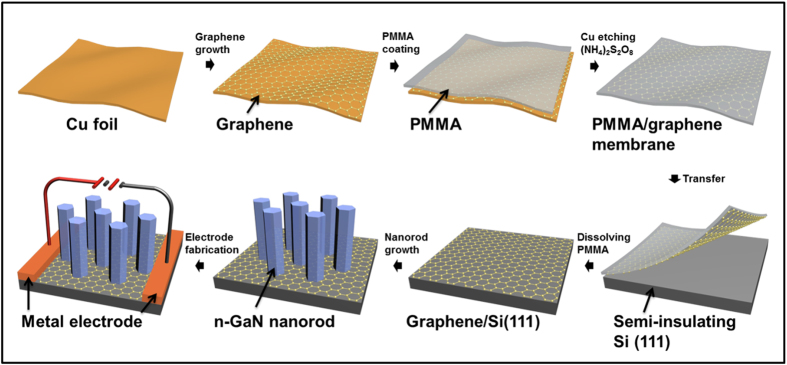
Growth and fabrication sequences of the n-GaN NR-graphene hybrid structure on a Si (111) substrate for photoconductive device applications.

**Figure 2:**
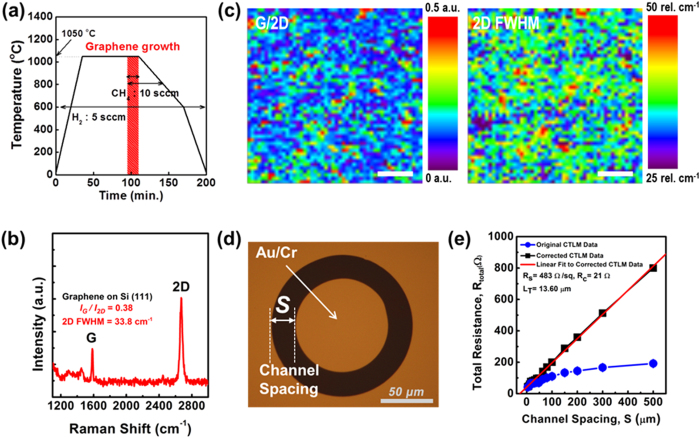
Characterization of monolayer graphene. (**a**) Schematic of the graphene synthesis process and comparison of the temperature profile vs. time. (**b**) Representative Raman spectrum of a monolayer graphene film grown on a Cu foil at 1050 °C and then transferred onto a Si (111) substrate. (**c**) Raman map of the G/2D-bands (left) and the FWHM of the 2D-band (right) of the graphene film transferred onto the Si substrate. The scale bars are all 15 μm. (**d**) An optical microscopy image of the patterned graphene layer on Si with an Au(35 nm)/Cr(5 nm) bilayer as ohmic contacts for CTLM measurements. (**e**) The total resistance measured using the CTLM method. The plot shows the total resistance as a function of channel spacing (S = 4–500 μm) after the correction factors were applied. The measured resistances (blue circles) are determined by the CTLM contact structure between the inner and outer contacts with various channel spacing. From the linear fit (red line), the following values are obtained: sheet resistance (*R*_*s*_), contact resistance (*R*_*c*_), and transfer length (*L*_*T*_). The error bars represent the standard deviation of the resistance in 5 different devices.

The fabrication sequences involved in the process used to synthesize n-GaN NR-graphene hybrid structures are shown in Fig. 1. A typical graphene growth procedure and the corresponding temperature profile are shown in Fig. 2a. The graphene synthesis process is discussed in detail in the Methods section. Figure 2b presents a representative Raman spectrum of graphene samples that were obtained at a temperature of approximately 1050 °C and then transferred onto a Si substrate. The spectrum exhibits two primary features: a G-band at approximately 1584 cm^−1^ and a 2D-band at approximately 2673 cm^−1^, which are typical peak positions for defect-free graphene^28^,^29^. The associated G/2D band and the full-width at half-maximum (FWHM) of the 2D-band maps in Fig. 2c illustrate the uniformity of the graphene films over large areas (approximately 490 μm^2^) that predominantly exhibit typical monolayer properties, as identified by the I_G_/I_2D_ ≤ 0.38 and the FWHM of the 2D band of ≤38 cm^−1^. Chu *et al.*^29^ have reported the formation of a graphene layer over SiO_2_/Si (100) with a G-band/2D-band peak intensity ratio ≤0.4, whereas Kwak *et al.*^30^ have reported a ratio of approximately 0.5. In this study, Raman spectroscopy revealed further improvement in the quality and uniformity of the monolayer graphene (with a peak intensity ratio I_G_/I_2D_ ≈ 0.38) grown using the CVD process and then transferred to a Si (111) substrate. To our knowledge, this study represents the finest graphene layer transferred directly to a Si substrate reported thus far^29^,^30^. The electrical properties of the graphene films that were transferred onto Si substrates were evaluated using the circular transfer length method (CTLM) structure, as illustrated in Fig. 2d. Figure 2e shows the measured resistances (blue circles) as a function of the channel spacing *S* from 4 to 500 μm. A geometry correction was extracted by fitting the measured resistances to the following equation^31^,^32^,^33^:
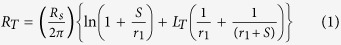


Here, *R*_*T*_ is the total resistance between the channel spacing of the contact pads, *R*_*s*_ is the sheet resistance of the graphene films, *r*_1_ is the radius of the inner circular contact, *S* is the channel spacing of the contact pads, and *L*_*T*_ is the transfer length. From the linear fit (red line), the estimated sheet resistance and transfer length of the graphene films were *R*_*s*_ ≈ 483 ohm/sq and *L*_*T*_ ≈ 13.60 μm, respectively, implying that highly crystalline monolayer graphene films were transferred onto the Si substrate.

### Excellent crystallinity of n-GaN NR-graphene hybrid structure

**Figure 3:**
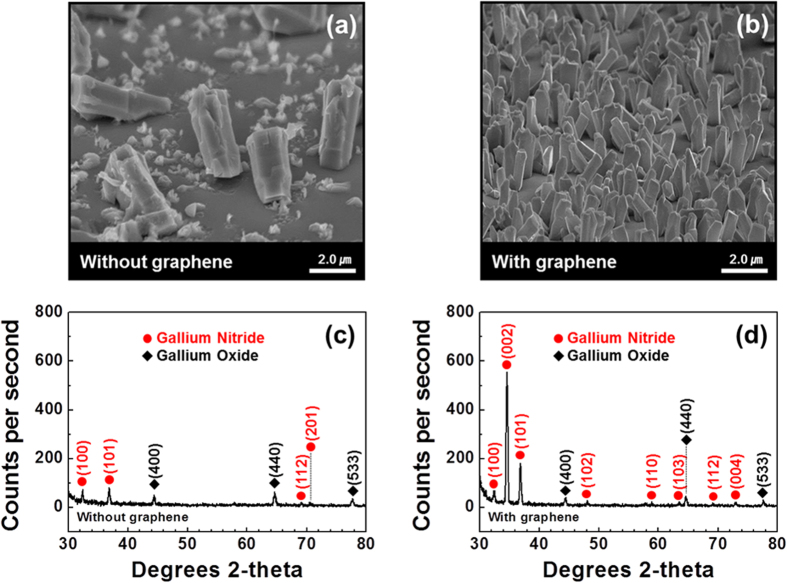
Tilt-view field-emission scanning electron microscopy (FE-SEM) images and XRD study of GaN NRs without and with a graphene layer. (**a**) FE-SEM image of low-density n-GaN NRs grown on a Si (111) substrate without the graphene layer. (**b**) Highly dense n-GaN NRs grown on monolayer graphene on a Si substrate. (**c**) The XRD pattern reveals the absence of n-GaN NRs along the (002) or *c*-plane when grown directly on the Si substrate. (**d**) A strong (002) peak is observed in the XRD pattern of the n-GaN NRs with the introduction of single-layer graphene on a Si substrate.

Figure 3a,b show the surface morphologies of GaN nanorods grown on Si substrates without and with a graphene layer, respectively. The monolayer graphene clearly provided a smooth surface for the growth of denser NRs with the prominent presence of individual NRs (Fig. 3b) and without further formation of structural defects. In addition, the graphene layer actually helped improve the material quality of the n-GaN NRs, as evident from the single-crystal X-ray diffraction (XRD) patterns (Fig. 3c,d). Figure 3c shows four XRD peaks for the GaN NRs centered along the (100), (101), (112) and (201) planes when grown without the graphene layer; however, no GaN NRs were grown along the (002) or *c*-plane. With the integration of a graphene layer, the XRD peaks indicated the existence of GaN NRs grown along the *c*-plane, i.e., along the (002) and (004) planes (Fig. 3d); the strong presence of the (002) plane confirmed the improvement in the crystallinity and material quality of the properly grown n-GaN NRs. The XRD results matched those of GaN and Ga_2_O_3_ listed in the Joint Committee on Powder Diffraction Standards (JCPDS) cards No. 50-0792 and 20-0426, respectively.

**Figure 4:**
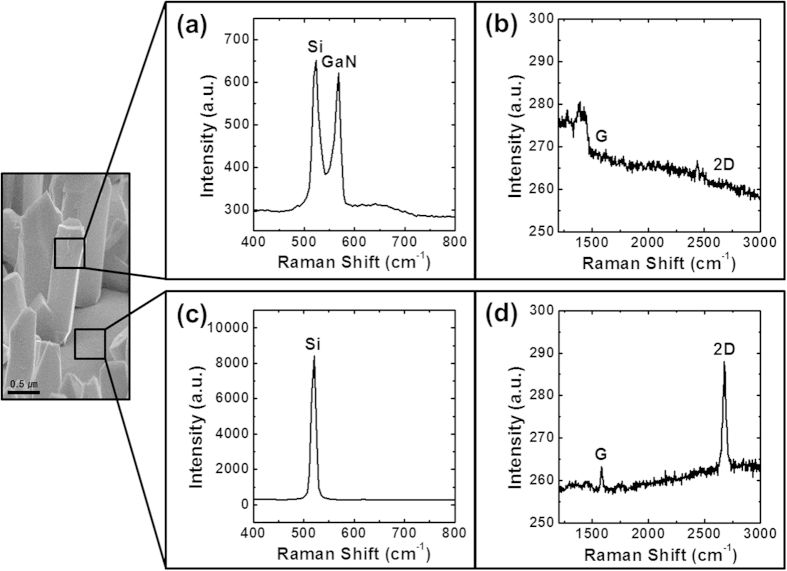
Raman spectroscopy performed on a nanorod body and the empty space between NRs. (**a**) The Raman spectrum shows peaks for both the Si substrate and the n-GaN NRs at approximately 520 cm^−1^ and 567 cm^−1^, respectively, when collected on a single NR. (**b**) The presence of weaker G- and 2D-bands was observed for graphene on the GaN NR body. (**c**) A high-intensity Si substrate peak was observed in the Raman spectrum of the empty space between nanorods. (**d**) Strong peaks of the G- and 2D-bands for graphene at approximately 1584 cm^−1^ and 2673 cm^−1^, respectively, were observed in the Raman spectrum of the empty spaces between GaN NRs, demonstrating that the graphene layer remained intact even after the growth of GaN NRs.

Additionally, when Raman spectroscopy was performed for an individual n-GaN NR, the Raman spectra revealed peak positions for the Si substrate and GaN nanorods at approximately 520 cm^−1^ and 567 cm^−1^, respectively (Fig. 4a); however, the spectrum of the nanorod body exhibited weak G-band and 2D-band peaks associated with graphene (Fig. 4b). The Raman spectrum of the empty space between the nanorods, as shown in Fig. 4c,d, exhibited strong peaks attributable to the Si substrate and the G-band and 2D-band peaks associated with graphene, respectively. Prominent G- and 2D-bands of higher intensity (compared to those in Fig. 4b) were observed at peak positions of approximately 1584 cm^−1^ and 2673 cm^−1^, respectively, in the spectrum of the graphene layer (Fig. 4d)^29^. This finding provided clear evidence that, even after the growth of n-GaN NRs on the monolayer graphene, the physical existence and material properties of the graphene layer were not affected and the graphene layer was not damaged or impaired. Even though the Si substrate peak was observed in the Raman spectrum of the nanorod body (Fig. 4a), this peak was much weaker compared to the Si peak in Fig. 4c.

**Figure 5:**
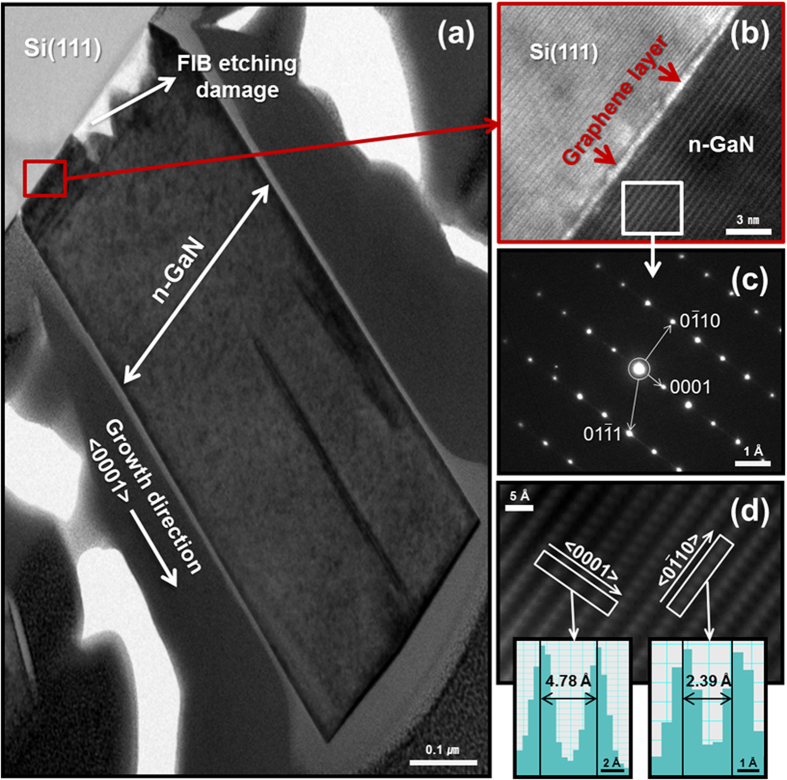
HR-TEM study for the hybrid structure. (**a**) HR-TEM image of an n-GaN NR grown in the <0001> direction on a graphene layer on a Si (111) substrate. (**b**) The high-magnification lattice image captured during HR-TEM analysis confirmed the presence of uniformly grown monolayer graphene on the Si substrate and the growth of defect-free n-GaN NRs over the graphene layer. (**c**) SAED pattern taken along the <11
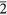
0> zone axis on n-GaN NR showing (0001), (0
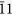
0), and (01
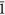
1) diffraction spots. (**d**) IFFT lattice images were useful for measuring the interplanar spacings of approximately 4.78 and 2.39 Å for <0001> and <0
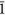
10> planes on NR segments, respectively.

High-resolution transmission electron microscopy (HR-TEM) images showed that the n-GaN NRs were uniformly grown along the *c*-plane, <0001> direction (Fig. 5a). To verify the nanorod-graphene hybrid heterostructure, a high-magnification lattice image was recorded. The n-GaN NR-graphene hybrid structure was clearly observed, and single-layer graphene was observed to exist on the Si substrate (confirmed by Raman spectroscopy in Fig. 2c); in addition, a defect-free n-GaN NR was observed to be overgrown on the graphene layer, as shown in Fig. 5b. The thickness of the graphene layer was almost uniform throughout the substrate and was determined to be less than 5 Å. The possibility of growing a well-defined 5 Å graphene layer and its prominent existence suggest that the proposed hybrid structure will exhibit high performance. A representative selected-area electron diffraction (SAED) pattern taken along the <11
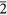
0> zone axis on n-GaN NR is presented in Fig. 5c, where (0001), (0
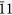
0), and (01
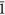
1) diffraction spots are present^25^,^34^. The clear diffraction spots indicate that the SAED pattern was of a high-quality hexagonal structure of a GaN NR. No extended defects, such as misfit dislocations and stacking faults, were observed in the n-GaN NRs. A clearer lattice construction of the n-GaN NR is shown in Fig. 5d, which was acquired from inverse fast-Fourier-transform (IFFT) imaging. The interplanar spacings of the <0001> and <0
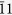
0> planes on the NR structure, as measured from TEM images, were approximately 4.78 and 2.39 Å, respectively, as shown in Fig. 5d.

**Figure 6:**
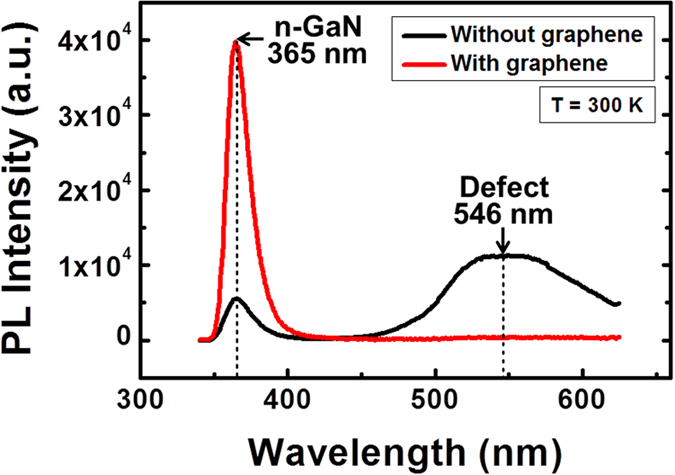
Comparison of room-temperature photoluminescence spectra. Emission from n-GaN NRs at 365 nm along with defect-related broad emission at approximately 546 nm when the NRs were directly grown on a Si (111) substrate (black line). The defect-related PL emission was well suppressed because of the integration of GaN NRs with the monolayer graphene (red line).

Figure 6 compares the photoluminescence (PL) spectra of n-GaN NRs grown on Si (111) substrates with (red line) and without (black line) a graphene layer. Both of the PL spectra show a band-edge emission at approximately 365 nm, corresponding to the n-GaN NR structures^34^. We also observed an emission as a broad band at approximately 546 nm in the spectrum of the n-GaN NRs grown on the Si substrate without graphene. The appearance of the broad emission at approximately 546 nm is associated with vacancy and structural defects^34^. However, in the spectrum of the n-GaN NRs grown on the graphene layer on the Si substrate, this broad emission originating from structural defects was not observed; i.e., the junction of the n-GaN NR-graphene heterostructure is almost strain-free. The defect-free growth of n-GaN NRs on graphene, as revealed by HR-TEM images (Fig. 5b), confirms this observation. The FWHM of approximately 20 nm of the PL peak corresponding to the n-GaN NRs grown without a graphene layer was reduced to approximately 17 nm with the introduction of the graphene layer; this observation confirms that the graphene layer not only acted as an obstruction for forming structural defects but may have also improved the uniformity and material quality of the n-GaN NRs. The improvement of the material quality of the n-GaN NRs can also be correlated with the XRD peaks corresponding to n-GaN NRs in Fig. 3d, where the intensity of the (002) peak in the XRD pattern of the n-GaN NRs was strong when a graphene layer was introduced. Therefore, on the basis of the results of our study, it is likely that defect-free n-GaN NR-graphene hybrid structures grown on Si substrates will be suitable for use in cost-effective, compact and efficient photoconductive device applications.

### Superior photoconductivity of the n-GaN NR-graphene hybrid devices

**Figure 7:**
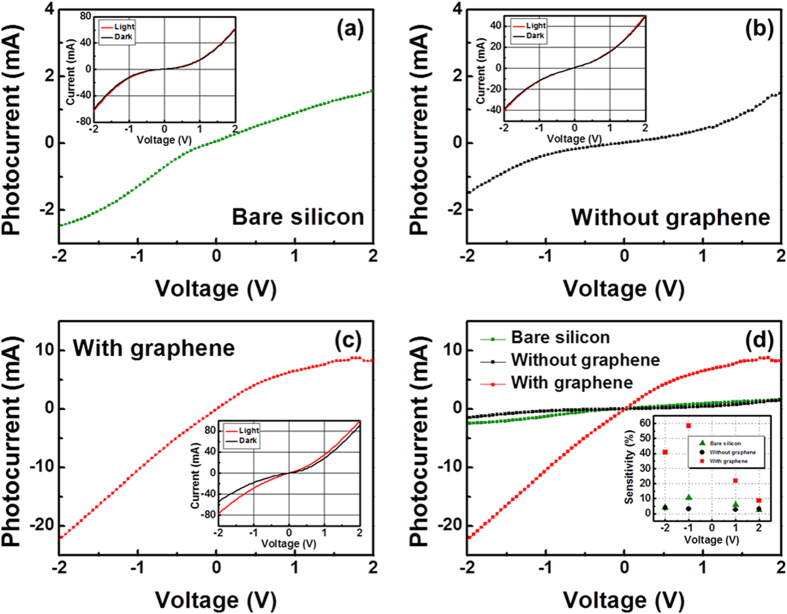
Photocurrent as a function of bias voltages between the metal electrodes. (**a**) Photocurrent from a bare semi-insulating Si (111) substrate. (**b**) Photocurrent from a heterostructure of n-GaN NRs grown on a Si substrate without any graphene layer. (**c**) The n-GaN NR-graphene hybrid structure on a Si substrate exhibits an increase in photocurrent. The insets of Figs. 7a,b,c show the I–V characteristics of photoconductive channels with (red line) and without (dark line) a light source. (**d**) The photocurrent increased almost nine fold for the n-GaN NR-graphene hybrid structure compared to that of the heterostructure without a graphene layer; the inset shows the variation of the sensitivity with bias voltages for the three types of channels under study.

The photocurrent behavior of the bare semi-insulating Si (111) substrate during photoconductivity measurements is shown in Fig. 7a, whereas Fig. 7b shows the photoconduction between n-GaN NRs on a Si substrate without a graphene layer. The photoconductivity of the channels based on the n-GaN NR-graphene hybrid structures on Si substrates is shown in Fig. 7c. The photocurrent I_P_ is defined as (I_max_ 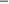 I_dark_) at a specific bias voltage^2^,^35^. The insets of Fig. 7a,b,c depict typical I–V characteristics of photoconductive channels with (red line) and without (dark line) a light source. Figure 7d shows the almost nine fold enhancement in the photocurrent for the n-GaN NR-graphene heterostructure when these three types of channels were compared. The inset of Fig. 7d shows the variation of the sensitivity^2^,^35^ (I_P_/I_dark_) with bias for the bare Si substrate and for n-GaN NRs on the same substrate without and with a graphene layer; the sensitivity was estimated to be approximately 10%, 4%, and 60%, respectively, at a bias voltage of 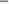
1.0 V. The degradation in sensitivity for the n-GaN NRs grown on the Si substrate without a graphene layer might be attributable to the presence of growth-related structural defects, as already established on the basis of the photoluminescence results (Fig. 6). The lower densities of the GaN NRs, as the field-emission scanning electron microscopy (FE-SEM) image illustrates (Fig. 3a), might be the additional cause of this low sensitivity for the same sample. With the introduction of a graphene monolayer, the absence of structural defects (Fig. 6), enhanced density (Fig. 3b) and improved material quality (Fig. 3d) of the GaN NRs contributed to the increase in sensitivity to 60%.

**Figure 8:**
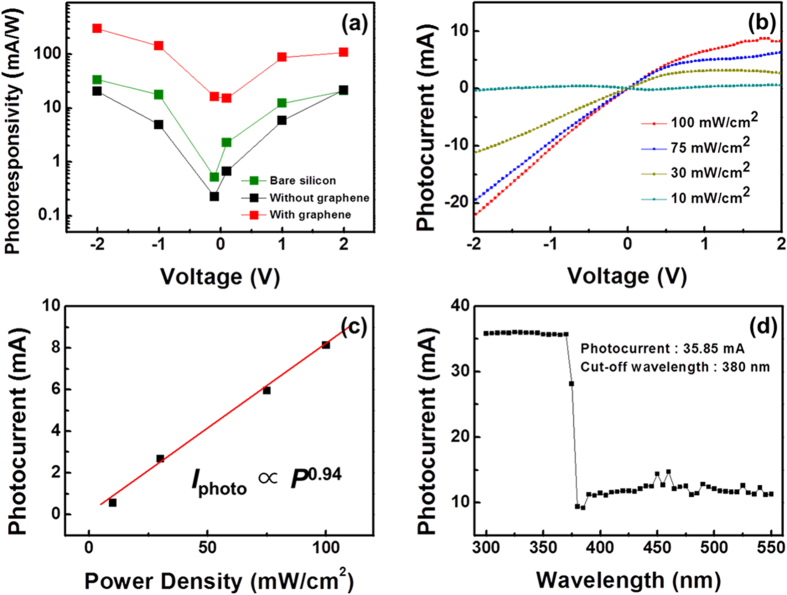
Photoresponsivity and cut-off wavelength measurements. (**a**) Variation of photoresponsivity with bias voltages for three types of channels: a bare semi-insulating Si (111) substrate and n-GaN NRs grown on the same substrate with and without a graphene layer. (**b**) Using various optical power densities of the light source, the photocurrent at different bias was measured for the n-GaN NR-graphene hybrid structure. (**c**) The photocurrent I_p_ measured at various optical power densities increased linearly, which was attributed to a reduced density of trap levels in the photoconductive channel; the exponent x in the power law has a high value of approximately 0.94. This measurement was performed with the bias fixed at 2.0 V. (**d**) The spectral photoresponse measurement reveals a cut-off wavelength of 380 nm for the n-GaN NR-graphene hybrid structure.

The same behavior of the photocurrent and sensitivity was repeated when the photoresponsivity of these three channels was compared (Fig. 8a). The photoresponsivity was estimated as I_P_/(Illuminated area × Power density of light)^2^,^35^. For our study, we used a maximum power density of 100 mW/cm^2^ and maintained the same dimensions of the illuminated area for all of the channels. At a bias of 2.0 V, the absolute photoresponsivity was calculated to be 20, 21, and 106 mA/W, respectively, for the bare Si substrate, n-GaN NRs grown without graphene and n-GaN NRs with graphene. As Fig. 3b previously demonstrated, a greater density of GaN NRs resulted from the underlying graphene layer. A larger responsivity was therefore evident for the hybrid structure because of both the reduced empty space between individual NRs and the larger photoconductive area in the channel. Photoresponsivity analysis again indicated that the n-GaN NR-graphene hybrid structure was always a better option as a photoconductive channel compared with GaN NRs directly grown on a Si substrate. Hyungwoo Lee *et al.*^2^ established that graphene could be used as a photoresponsive channel for graphene-CdS NR hybrid structures and, thus, that the photoresponsivity of the structures could be enhanced. We applied the same concept for the n-GaN NR-graphene hybrid structure under study. Graphene exhibits excellent electrical conductivity^2^,^14^,^36^ even though the lifetime of photogenerated carriers is very short in monolayer graphene^2^,^37^,^38^. Thus, a single layer of graphene can serve as a highly efficient transport medium of charge carriers between n-GaN NRs and metal electrodes.

Different characterization methods have already been used to establish that the material quality of n-GaN NRs is improved when grown on monolayer graphene rather than directly on a Si substrate. To validate this fact through the electrical behavior of the channel, an optical-power-dependent study of the photocurrent was performed. When the photocurrent was measured with various optical power densities of the light source for the n-GaN NR-graphene heterostructure, the photocurrent was observed to increase with increasing incident optical power density (Fig. 8b). Various photocurrent values at a bias of 2.0 V were plotted for this channel against the optical power density (Fig. 8c) and fitted by a simple power law, I_p_ ∞ P^x^ 2,35,39, where P is the incident optical power of the white light source and the exponent x determines the density of trap levels between the Fermi level and the conduction band of the n-GaN NRs in the photoconductive channel under study. These trap levels are responsible for the absorption of photogenerated carriers and result in suppression of the photocurrent in the channel. With a lower density of trap levels, even at low incident power, photogenerated carriers should participate in the photocurrent without being absorbed by the trap levels. Thus, the photocurrent I_p_ would increase linearly with increasing optical power when the trap level density is lower. To support this fact, the exponent x should be approximately 1 in the power law. If the photoconductive channel possesses a high density of trap levels, x should be significantly smaller than 1. When the photocurrent values were fitted in Fig. 8c, the photoconductive channels showed x ≈ 0.94, which is larger than any value reported thus far for nanorod-graphene hybrid structures^2^. The evidence of minimal defects or trap states in the channel reveals why this MOCVD-grown n-GaN NR-graphene hybrid structure on Si is ideal for use in cost-effective, highly efficient photoconductive device applications. The photocurrent values, which are associated with the spectral response, were measured to be almost constant at approximately 36 mA at wavelengths up to an abrupt cut-off wavelength of 380 nm (Fig. 8d). For light of wavelengths longer than 380 nm, the photons did not possess sufficient energy to generate photocarriers to contribute to the photocurrent. Thus, the photocurrent values were measured to be approximately 10 mA at wavelengths longer than 380 nm for this n-GaN NR-graphene hybrid structure (Fig. 8d).

## Discussion

This study demonstrates the superior photoconductive behavior of an n-GaN NR-graphene hybrid device structure synthesized on a Si (111) substrate using a high-quality graphene transfer method and the relatively low-temperature MOCVD process with a high V/III ratio. Raman spectroscopy and HR-TEM images confirmed the presence of monolayer graphene highly matched with n-GaN NRs in the hybrid structure. The excellent electrical conduction properties of the graphene monolayer transferred on Si (111) not only affected the growth of defect-free, highly dense n-GaN NRs but also improved the material quality. As a result, the n-GaN NR-graphene hybrid structure under study represents the superior characteristics of an ultraviolet photoconductive device with extremely low carrier trap density. Moreover, the synthesis of the hybrid structure directly on a Si substrate by the growth of n-GaN NRs without using metal-catalyst or droplet seeds added special significance. To our knowledge, such simple, cost-effective and highly efficient ultraviolet photoconductive devices fabricated using highly matched n-GaN NR-graphene hybrid structures are reported here for the first time.

## Methods

### CVD growth of graphene and transfer method

First, a Cu foil (25 μm thick, 99.8%, Alfa Aesar) was electrochemically polished in H_3_PO_4_ (85%) solution to obtain a flat and smooth Cu foil surface with a root-mean-square surface roughness of less than 15 nm (in the scanning area of 100 μm × 100 μm). After the Cu foil was loaded into a low-pressure chemical vapor deposition (LP-CVD) system, it was heated to a process temperature of 1050 °C and maintained for 60 min under 5 sccm of H_2_ for *in situ* surface cleaning. A monolayer graphene film was then grown for 15 min under a CH_4_/H_2_ mixture (10 and 5 sccm, respectively) with a reactor pressure of approximately 90 mTorr. After graphene growth, the chamber was rapidly cooled to room temperature with a flow of H_2_. Following graphene growth, the graphene film was transferred onto a Si substrate for further investigation. In all of our growth experiments, monolayer graphene was observed to grow on both sides of the Cu foil; therefore, the backside of the Cu foil was pre-treated with oxygen plasma to remove the undesired graphene film. To transfer the graphene film to a Si substrate, poly(methyl methacrylate) (PMMA) was spin-coated onto the graphene grown on Cu foil, and the PMMA/graphene/Cu foil was then cured on a hot plate at 180 °C for 1 min. The Cu foil was then etched from the PMMA/graphene/Cu foil assembly using an aqueous solution of (NH_4_)_2_S_2_O_8_. The PMMA/graphene assembly was subsequently washed with deionized water, transferred onto a semi-insulating Si (111) substrate and dried. The PMMA was then removed using acetone.

### Synthesis of highly matched n-GaN NR-graphene hybrid structure using MOCVD

n-GaN nanorods were grown on the graphene-transferred Si (111) substrate using a relatively low-temperature MOCVD process and a high V/III ratio without the formation of any metal-catalyst or droplet seeds. The precursors for gallium and nitrogen were trimethylgallium (TMGa) and ammonia (NH_3_), respectively; silane (SiH_4_) gas was used for n-type doping, and hydrogen was used as the carrier gas. To grow n-GaN nanorods, TMGa, NH_3_ and SiH_4_ were introduced into the reactor chamber for 45 min at flow rates of 0.2 sccm, 3.0 SLM and 10 sccm, respectively (V/III ratio = 15,000). To protect the graphene layer from thermal damage during the growth of the n-GaN nanorods, the growth temperature was fixed at 870 °C, which is relatively low compared to the usual temperatures of greater than 1000 °C^15^. Finally, Au/Ni metal electrodes were formed on the graphene layer to evaluate the photocurrent, photoresponsivity and cut-off wavelength.

### Characterization of the hybrid structure and fabricated device

Raman spectroscopy was performed using a WiTec Alpha 300R M-Raman system; the excitation wavelength was 532 nm. The results of the Raman study were used to confirm the existence of highly ordered graphene films. The surface morphology of the n-GaN NRs was analyzed using FE-SEM. A Hitachi S-7400 system was used for the FE-SEM study; it was operated at 15 kV and at a 13° tilt-view. Single-crystal XRD measurements were performed using a Rigaku diffractometer equipped with a Cu-Kα radiation source. The morphology of the n-GaN NR-graphene heterostructures was further revealed by cross-sectional HR-TEM. A JEOL JEM 2010 system operated at 200 kV was used for the HR-TEM study. Samples of the hybrid structure were prepared by being coated with platinum using a dual-beam focused ion beam (FIB, Quanta 3D FEG) technique with a beam current of 65 nA and a resolution of 7 nm at 30 kV. The optical properties of the n-GaN NRs were investigated by photoluminescence (PL) spectroscopy at room temperature using the 325 nm line of a He-Cd laser as an excitation source. For photoconductivity measurements of the fabricated ultraviolet photoconductive device, we utilized a solar simulator (McScience Lab 100) as a light source. This light source generated white light with a maximum power density of 100 mW/cm^2^. Spectral photoresponse measurements of the fabricated device were performed with a xenon arc lamp (300 W) within the range of 300–550 nm. A monochromator (Oriel Cornerstone 130) was used to provide the monochromatic light incident on the channel.

## Additional Information

**How to cite this article**: Kang, S. *et al.* Ultraviolet photoconductive devices with an n-GaN nanorod-graphene hybrid structure synthesized by metal-organic chemical vapor deposition. *Sci. Rep.* **5**, 10808; doi: 10.1038/srep10808 (2015).
